# Longitudinal whole-brain atrophy and ventricular enlargement in nondemented Parkinson's disease

**DOI:** 10.1016/j.neurobiolaging.2017.03.012

**Published:** 2017-07

**Authors:** Elijah Mak, Li Su, Guy B. Williams, Michael J. Firbank, Rachael A. Lawson, Alison J. Yarnall, Gordon W. Duncan, Brit Mollenhauer, Adrian M. Owen, Tien K. Khoo, David J. Brooks, James B. Rowe, Roger A. Barker, David J. Burn, John T. O'Brien

**Affiliations:** a Department of Psychiatry, University of Cambridge, Cambridgeshire, UK; b Wolfson Brain Imaging Centre, University of Cambridge, Cambridgeshire, UK; c Institute of Neuroscience, Newcastle University, Newcastle upon Tyne, UK; d Medicine of the Elderly, Western General Hospital, Edinburgh, UK; e Paracelsus-Elena-Klinik, Kassel, Germany; f University Medical Center Goettingen, Institute of Neuropathology, Goettingen, Germany; g Brain and Mind Institute, University of Western Ontario, London, Canada; h Department of Psychology, University of Western Ontario, London, Canada; i Menzies Health Institute, Queensland and School of Medicine, Griffith University, Gold Coast, Australia; j Division of Neuroscience, Imperial College London, London, UK; k Institute of Clinical Medicine, Aarhus University, Aarhus, Denmark; l Department of Clinical Neurosciences, University of Cambridge, Cambridge, UK; m Medical Research Council, Cognition and Brain Sciences Unit, Cambridge, UK; n Behavioural and Clinical Neuroscience Institute, University of Cambridge, Cambridge, UK; o John van Geest Centre for Brain Repair, University of Cambridge, Cambridge, UK

**Keywords:** Imaging, Parkinson's disease, Dementia, MRI, Longitudinal, Neurodegeneration

## Abstract

We investigated whole-brain atrophy and ventricular enlargement over 18 months in nondemented Parkinson's disease (PD) and examined their associations with clinical measures and baseline CSF markers. PD subjects (n = 100) were classified at baseline into those with mild cognitive impairment (MCI; PD-MCI, n = 36) and no cognitive impairment (PD-NC, n = 64). Percentage of whole-brain volume change (PBVC) and ventricular expansion over 18 months were assessed with FSL-SIENA and ventricular enlargement (VIENA) respectively. PD-MCI showed increased global atrophy (−1.1% ± 0.8%) and ventricular enlargement (6.9 % ± 5.2%) compared with both PD-NC (PBVC: −0.4 ± 0.5, *p* < 0.01; VIENA: 2.1% ± 4.3%, *p* < 0.01) and healthy controls. In a subset of 35 PD subjects, CSF levels of tau, and Aβ42/Aβ40 ratio were correlated with PBVC and ventricular enlargement respectively. The sample size required to demonstrate a 20% reduction in PBVC and VIENA was approximately 1/15th of that required to detect equivalent changes in cognitive decline. These findings suggest that longitudinal MRI measurements have potential to serve as surrogate markers to complement clinical assessments for future disease-modifying trials in PD.

## 1 Introduction

Up to 80% of Parkinson's disease (PD) patients eventually develop dementia (PD-D) (Aarsland et al., 2003). However, the pathophysiological substrates of cognitive dysfunction leading up to the demented state remain only partially understood (Nombela et al., 2014, Williams-Gray et al., 2009, Winder-Rhodes et al., 2013, Winder-Rhodes et al., 2015, Yarnall et al., 2013). In parallel with a recent shift toward early interventions and the prospect of disease-modification (e.g., immunotherapy and apomorphine) in nondemented PD (Yarnall et al., 2015), there is an urgent need to identify surrogate markers to track disease progression and perform risk-stratification to improve patient enrollment in clinical trials.

At present, psychometric tests and severity rating scales (i.e., Unified Parkinson's Disease Rating Scale, UPDRS) are the *de facto* standard for evaluating disease progression in PD. There is however increasing interest in adopting longitudinal neuroimaging techniques as adjunctive markers of disease progression with the expectation that MRI measurements may provide better sensitivity and precision than standard clinical measures (Jack et al., 2003, Nestor et al., 2008, Schott et al., 2005). In this regard, advances in neuroimaging analyses have contributed to the validation of whole-brain atrophy rates (Smith et al., 2002) as sensitive markers of disease progression in mild cognitive impairment (MCI; Sluimer et al., 2010), frontotemporal dementia (Knopman et al., 2009), and Alzheimer's disease (AD; Fox and Freeborough, 1997, Mak et al., 2015b). In addition, ventricular enlargement has emerged as another viable surrogate but not nonspecific marker of neurodegeneration in MCI and AD (Ferris et al., 2009, Jack et al., 2004, Nestor et al., 2008). Instead of a mere proxy of widespread tissue loss, ventricular enlargement has been linked to a broad range of cognitive and memory deficits, reduced brain reserve against neurodegeneration (Cavedo et al., 2012), and decreased survival in dementia (Olesen et al., 2011).

Power calculations in MCI and AD have consistently shown that whole-brain atrophy and ventricular enlargement would require far smaller sample sizes (approximately 3–10 times reduction) compared with cognitive tests to show differences from controls (Jack et al., 2004, Ridha et al., 2008). This has significant implications on the design of early-intervention and secondary prevention trials that are often hampered by subtle disease-related decline in the prodromal stages (i.e., weak effect sizes) and greater uncertainty that participants are on course for developing dementia (i.e., greater variance in measurements). As a result, these trials would require very long follow-up duration as well as large samples to detect any disease-modifying effects.

Therefore, it is surprising that there are only limited studies investigating the utility of whole-brain atrophy and ventricular enlargement in PD. While increased whole-brain atrophy rates have been reported in PD-D compared with controls (Burton et al., 2005), it remains to be established if MRI-derived measurements of global atrophy are sensitive to changes in a prodromal stage such as PD-MCI, and whether these measurements are feasible in a clinical trial targeting cognitive symptoms in PD. Ventricular enlargement has also been less studied in PD (Camicioli et al., 2011) despite its associations with both motor and cognitive impairment (Apostolova et al., 2012).

Levels of CSF markers have been shown to be promising candidate markers in AD (Blennow and Hampel, 2003, Frisoni et al., 2010) and more recently in PD (Kang et al., 2016). Elucidating the potentially unique role of each CSF marker in the later events of neurodegeneration (i.e., structural atrophy) will have important implications for informing strategies targeting the underlying protein pathologies. In a pooled sample of PD patients, both CSF T-Tau and Aβ levels have been cross-sectionally associated with lateral ventricular size (Beyer et al., 2013), whereas there are only limited studies investigating the involvement of CSF markers and progressive atrophy in PD (Compta et al., 2013).

To address the aforementioned gaps in the literature, we undertook a new study with 3 main objectives: (1) to investigate the suitability of global longitudinal measurements of brain volume (whole-brain atrophy and ventricular enlargement) to monitor disease progression over 18 months in newly-diagnosed PD patients; (2) to evaluate the relationships between baseline CSF markers of neurodegeneration (α-synuclein, tau-protein) and structural changes on imaging using tensor-based morphometry; and (3) to assess the impact of using MRI measurements on future clinical trials in nondemented PD patients by estimating the sample sizes needed to detect a 20%–50% reduction in whole-brain atrophy, ventricular enlargement, and global cognition.

## 2 Method

### 2.1 Participants

The Incidence of Cognitive Impairment in Cohorts with Longitudinal Evaluation-PD is a longitudinal observational study with 2 centers (Newcastle and Cambridge) to understand the disease mechanisms underlying the evolution of PD-D from disease onset (Yarnall et al., 2014). Patients were recruited from community and outpatient clinics in the North East of England. In this study, we included PD subjects (n = 104) and healthy controls (n = 38) who completed baseline and follow-up clinical and T1 MRI imaging at 18 months. PD was diagnosed according to the UK Brain Bank criteria by a movement disorders specialist (Hughes, 2002). Full inclusion and exclusion criteria have been previously described (Yarnall et al., 2014); patients were excluded at baseline if they had a clinical diagnosis of PD-D or scored <24 on the Mini–Mental State Examination. The study was approved by the Newcastle and North Tyneside Research Ethics Committee. All subjects provided written informed consent.

### 2.2 Clinical and neuropsychological assessment

Clinical assessments were performed by trained examiners and included a standardized neurological examination and rating disability with the Movement Disorders Society (MDS; UPDRS III; Goetz et al., 2008), and Hoehn and Yahr (H&Y) staging (Hoehn and Yahr, 2001). In accordance with MDS Task Force recommendations (Litvan et al., 2012), 5 cognitive domains were assessed: attention was measured using the Cognitive Drug Research computerized battery (Wesnes et al., 2002). Mean response times of simple reaction time, choice reaction time, and digit vigilance were summed to produce a Power of Attention score. Digit vigilance accuracy was also evaluated as part of this domain. Memory was assessed with pattern recognition memory, spatial recognition memory, and paired associates learning from the computerized Cambridge Neuropsychological Test Automated Battery (Fray and Robbins, 1996). Executive function was determined using the modified “One Touch Stockings” (OTS) version of the Tower of London task from the Cambridge Neuropsychological Test Automated battery, phonemic fluency (words beginning with “F” in 1 minute) and semantic fluency (animals in 90 seconds). The pentagon copying item of the MMSE was graded using a modified 0 to 2 rating scale as a measure of visuospatial function (Williams-Gray et al., 2009). Language domain was assessed using the naming (0–3) and sentence (0–2) subsets of the MoCA test. All participants were assessed while they were on their usual dopaminergic medication at baseline and 18 months. Levodopa equivalent daily dose (LEDD) value was calculated using the Tomlinson et al. formula (Tomlinson et al., 2010). Global cognitive function was assessed using the Mini–Mental State Examination (MMSE) (Folstein et al., 1975) and the Montreal Cognitive Assessment (MoCA; Dalrymple-Alford et al., 2010). As our schedule of neuropsychological tests preceded the introduction of the MDS criteria for PD-MCI, we used a modified MDS level II criteria as described previously (Lawson et al., 2016, Yarnall et al., 2014), in that only 1 test (i.e., pentagon copying) was specific to the visuospatial domain. A subject was diagnosed as PD-MCI if he or she performed 1.5 standard deviations (SDs) or more below appropriate norms (derived from controls) on at least 2 neuropsychological tests across 5 cognitive domains: attention, memory, executive function, language, and visuospatial function. To reduce the number of comparisons, we derived a composite Z-score for each domain based on the average of the Z-scores across the respective tests. Using this cut-off, 38 PD subjects were classified as PD-MCI, whereas the remaining 66 PD subjects were classified as PD-NC. We classified participants as amnestic (n = 26) or nonamnestic PD-MCI (n = 12) at baseline. Amnestic participants were impaired in at least 2 tests across the 5 domains with at least 1 impaired memory test, whereas nonamnestic PD-MCI participants did not have impaired memory.

### 2.3 Lumbar puncture

At baseline, lumbar puncture was performed on a subset of PD subjects (n = 35) between 8 and 10 AM after an overnight fast and while withholding PD medications as described (Yarnall et al., 2014). Samples were centrifuged (2000 g, 4 °C, 10 minutes) within 15 minutes of collection and frozen at −80 °C in polypropylene cryovials until analyzed for β-amyloid 1–42 and 1–40 (Aβ42 and Aβ40), T-Tau, P-Tau, and total α-synuclein levels. Based on previous evidence that the combined ratio of Aβ42/Aβ40 is a more precise marker of Aβ pathology than Aβ42 and Aβ40 alone (Janelidze et al., 2016, Koyama et al., 2012), we derived a ratio of Aβ42/Aβ40 for subsequent correlational analyses. The total PD sample with CSF measurements was stratified into low and normal Aβ42 groups using a median split (≤948 pg/mL).

### 2.4 MRI acquisition and image analyses

Subjects underwent baseline and repeat MR imaging after 18 months. Both MRI acquisitions were performed with the same 3T MRI system (Intera Achieva scanner, Philips Medical Systems, Eindhoven, Netherlands). The structural scans were acquired using a standard T1-weighted volumetric sequence covering the whole brain: 3D magnetization-prepared rapid gradient echo sequence, sagittal acquisition, echo time (TE) = 4.6 ms, repetition time (TR) = 9.6 ms, inversion time 1250 ms, flip angle = 8°, SENSE factor = 2, in-plane field of view 240 × 240 mm yielding a voxel size of 1.15 × 1.15 mm with slice thickness of 1.2 mm.

### 2.5 Estimation of longitudinal whole-brain atrophy

**Fig. 1:**
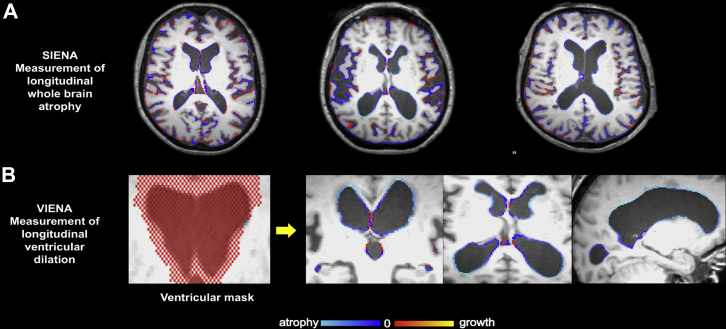
Illustration of imaging pipeline for whole-brain atrophy rates and ventricular enlargement over time. (A) Whole-brain atrophy as quantified by SIENA. Each image shows the changes between baseline and 18 months for each representative subject. Red indicates brain volume increase, whereas blue represents atrophy. (B) VIENA was used to calculate ventricular percentage changes between baseline and 18 months after a ventricular mask (red pixels) were registered to each individual MRI to identify ventricular edge displacements. Abbreviation: VIENA, ventricular enlargement. (For interpretation of the references to color in this figure legend, the reader is referred to the Web version of this article.)

Serial whole-brain atrophy was automatically estimated with SIENA, a commonly used software package for measuring longitudinal whole-brain atrophy that is distributed as part of the FSL imaging suite (http://fsl.fmrib.ox.ac.uk/fsl/fslwiki/). The technical details have been described (Smith et al., 2002, Smith et al., 2007). Briefly, for each individual subject, the baseline and follow-up brain images were aligned to each other using the skull images to constrain the registration scaling, and both brain images were then resampled into an intermediate halfway space with affine transformation. Next, tissue-type segmentation was carried out to locate brain/nonbrain edge points. The displacement of the follow-up brain image compared with the baseline was calculated as the edge-point displacement perpendicular to the surface. Finally, the mean edge displacement across the whole brain was converted into a global estimate of percentage brain volume change (PBVC) between the 2 time-points (Fig. 1). The quality of the registrations across time points, brain masks, and final outputs was then visually inspected while blinded to diagnostic group information, during which 5 subjects were excluded (1 healthy control, 2 PD-MCI, and 2 PD-NC). SIENA has been shown to have 0.5% brain volume accuracy in previous longitudinal studies (Smith et al., 2002).

### 2.6 Quantification of longitudinal ventricular enlargement

In addition, a recently validated technique (VIENA) was used to measure the percentage of ventricular volume change over 18 months. The technical details for this procedure have been described at length in the validation paper (Vrenken et al., 2014). In summary, the average edge displacement was calculated on edge points along the ventricular boundaries. This step made use of a ventricular mask, in MNI152 space, of a single MS patient with exceptionally large ventricles. The ventricular mask was then linearly registered to each individual MRI. Finally, the average ventricular edge displacement between both time-points was converted into a summary value (Fig. 1). This technique has been found to show a high agreement with manual measurements with a concordance correlation coefficient (CC_r_) >0.8 (Vrenken et al., 2014). The whole process took only 10–20 minutes per subject, rendering it practically convenient for large data sets.

### 2.7 Regional volumetric analysis

We performed a voxel-wise estimation of regional tissue change from the deformation field required to warp a subject's follow-up image to his/her baseline. We used the SPM output of divergence rate as the measure of volumetric expansion/contraction (Ashburner and Ridgway, 2013). The divergence rate images were spatially normalized and smoothed with an 8-mm Gaussian kernel. A voxel-wise general linear model was then used to investigate regional volumetric correlations with T-Tau and P-Tau, covarying for age, gender, and LEDD. Results were thresholded at *p* = 0.001 uncorrected for multiple comparisons, and clusters reported as significant at *p* < 0.05; family-wise error corrected.

### 2.8 Statistical analyses

Statistical analyses were performed with the STATA13 (http://www.stata.com/) software. The distribution of continuous variables was tested for normality using the Skewness-Kurtosis test and visual inspection of histograms. Parametric data were assessed using either T-tests or analysis of variance for continuous variables. For nonparametric data, Wilcoxon rank-sum or Kruskal-Wallis tests were used. χ^2^ tests were used to examine differences between categorical variables. Effect sizes are reported in terms of eta^2^ and Cohen's *D* where appropriate. Partial correlations with relevant covariates were performed to examine associations of imaging changes (whole-brain atrophy and ventricular enlargement) with cognitive/clinical measures and CSF markers. To determine whether the associations between CSF markers and neurodegeneration are affected by baseline amyloid burden, we classified the PD subjects into low and normal levels of CSF Aβ42 based on a median split. The mean concentration of CSF Aβ42 in the low group was 706.7 pg/mL, roughly in line with previous cut-off values of 700 pg/mL in AD (Lehmann et al., 2014). For each test statistic, a 2-tailed probability value of <0.05 was regarded as significant.

### 2.9 Sample size estimates for clinical trials

To investigate the impact of whole-brain atrophy and ventricular enlargement on clinical trial designs in nondemented PD, we performed sample size calculations with 80% power to detect a 20%–50% reduction in whole-brain atrophy, ventricular enlargement or MMSE (5% type I error, 2-tailed significant test *p* < 0.05). Sample size was calculated using the conventional equation in previous studies (Fox et al., 2000, Schott et al., 2010):

$$Sample\;size\;per\;arm=\frac{{\left(u+v\right)}^{2}\left(2\sigma^{2}\right)}{\Delta^{2}}$$

where σ^2^ denotes the variance of outcome measure estimated in the PD-MCI group, u = 0.84 to provide 80% power; v = 1.96 to test at the 5% significance level, Δ = estimated treatment effect = (0.2–0.5) × estimated whole-brain atrophy/ventricular enlargement/MMSE decline in the PD-MCI group across 18 months. Sample sizes were not derived for PD-NC since there were no significant differences in imaging outcomes compared with healthy controls.

## 3 Results

### 3.1 Sample characteristics

The demographic and clinical information for PD and control subjects are summarized in Table 1. PD-MCI subjects were significantly older than PD-NC (*p* = 0.001), although there were no significant differences in age and gender between PD-MCI and healthy controls, or between PD-NC and healthy controls. As expected, PD-MCI scored significantly lower on both MMSE and MoCA compared with PD-NC (*p* < 0.001) and healthy controls (*p* < 0.001).

**Table 1:** Demographics and clinical characteristics

Subject groups	HC	PD-NC	PD-MCI	*p*
*n*	37	64	36	
Age (y)	65.7 ± 7.2	62.8 ± 10.0	68.7 ± 8.7	0.01^b^
Age range	49.7–85.4	41.8–87.3	48.1–85.5	
Gender (male, %)	21, 56.8%	40, 63%	26, 72.2%	0.4^a^
Education (y)	13.9 ± 3.9	13.8 ± 3.6	11.3 ± 3.3	<0.001^f^
Disease duration (mo)		24.2 ± 4.7	24.7 ± 5.4	0.8^g^
LEDD (mg/d) baseline		144.5 ± 111.7	248.6 ± 158.1	0.001^g^
H&Y baseline		1.9 ± 0.7	2.1 ± 0.6	0.054^h^
H&Y follow-up		2.1 ± 0.6	2.2 ± 0.4	0.2^g^
UPDRS III baseline		25.0 ± 11.0	29.4 ± 10.5	0.051^h^
UPDRS III follow-up		31.4 ± 12.6	38.6 ± 10.0	0.001^g^
MMSE baseline	29.4 ± 1.0	29.1 ± 0.8	28.1 ± 1.4	<0.001^b^^,^^c^^,^^d^, 0.4^e^
MMSE follow-up	29.6 ± 0.9	29.2 ± 1.0	27.4 ± 1.9	<0.001^b^^,^^c^^,^^d^, 0.2^e^
MoCA baseline	27.6 ± 2.2	26.9 ± 2.4	22.9 ± 3.9	<0.001^b^^,^^c^^,^^d^, 0.4^e^
MoCA follow-up	27.9 ± 3.0	27.8 ± 2.1	24.1 ± 3.6	<0.001^b^^,^^c^^,^^d^, 0.9^e^
Cognitive domains^g^				
Memory		−0.2 ± 0.7	−2.0 ± 1.6	<0.001
Memory follow-up		−0.5 ± 1.0	−2.3 ± 1.5	<0.001
Attention		−0.1 ± 0.6	−1.6 ± 2.0	<0.001
Attention follow-up		−0.4 ± 0.9	−2.1 ± 2.1	<0.001
Executive		−0.2 ± 0.6	−1.2 ± 0.9	<0.001
Executive follow-up		0.02 ± 0.8	−1.2 ± 1.0	<0.001
Language		0.05 ± 0.7	−0.5 ± 1.0	<0.01
Language follow-up		0.1 ± 0.7	−0.3 ± 0.9	<0.01
Visuospatial		0.2 ± 0.5	−0.8 ± 2.1	<0.001
Visuospatial follow-up		−0.2 ± 1.2	−1.8 ± 3.0	<0.001

Values expressed as mean ± SD.

Key: GDS, Geriatric Depression Scale; H&Y, Hoehn and Yahr; MMSE, Mini–Mental State examination; MOCA, Montreal Cognitive Assessment; PD-MCI, Parkinson's disease with mild cognitive impairment; PD-NC, Parkinson's disease with no cognitive impairment; UPDRS III, Unified Parkinson's Disease Rating Scale, Part III.

a χA–Abbrev; PD-MCI, controls.

b ANOVA–Healthy controls, PD-NC, PD-MCI; Post hoc Tukey pairwise tests.

c PD-MCI versus healthy controls.

d PD-MCI versus PD-NC.

e PD-NC versus healthy controls.

f Kruskal-Wallis test.

g Wilcoxon rank-sum test–PD-NC and PD-MCI.

h Student *t* test–PD-NC and PD-MCI.

### 3.2 Comparisons of percentage brain volume change and ventricular enlargement

**Fig. 2:**
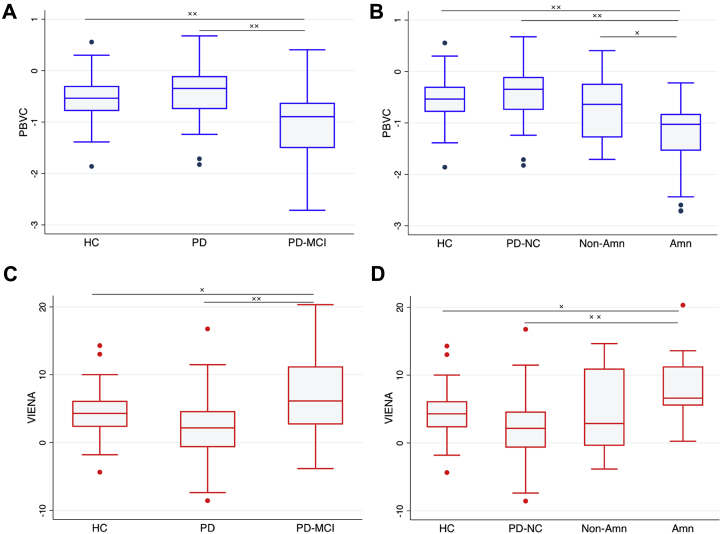
Box-and-whisker plots for comparisons of PBVC and ventricular enlargement for each group over 18 months. Negative % represents a decrease in volume over time. Differences between groups were assessed by using ANCOVA controlling for age, gender, education, inter-scan interval, and LEDD, with post hoc Tukey-Kramer tests. (A) Whole-brain atrophy was greater in PD-MCI compared with both PD-NC and HC (B) Only the amnestic subgroup of PD-MCI showed increased whole-brain atrophy compared with PD-NC, HC, and non-amnestic PD-MCI. (C, D) Ventricular enlargement was greater in PD-MCI compared with PD-NC and healthy controls, particularly in the amnestic subgroup of PD-MCI. Abbreviations: Amn, amnestic; HC, healthy controls; NON-amn, non-amnestic; PBVC, percentage brain volume change; PD-MCI, Parkinson's disease with mild cognitive impairment; PD-NC, Parkinson's disease with no cognitive impairment; VIENA, ventricular enlargement.

The extent of atrophy occurring between the baseline and follow-up scan was expressed as a negative percentage of PBVC (Fig. 2). ANCOVA revealed that PBVC was significantly different between groups [F(2,130) = 9.6, *p* < 0.01; eta^2^ = 0.13] after accounting for age, gender, education, and scan-intervals. Post hoc Tukey-Kramer tests revealed significantly greater percentage atrophy in PD-MCI compared with healthy controls and PD-NC groups (*p* < 0.01), although there were no differences in PBVC between PD-NC and controls (*p* = 0.99). After accounting for LEDD dosage, percentage atrophy was still significantly higher in PD-MCI compared with PD-NC (*p* < 0.01). Stratification of PD-MCI into amnestic (n = 25) and nonamnestic (n = 12) subgroups at baseline revealed that only the MCI-amnestic group showed increased whole-brain atrophy compared with controls and PD-NC (*p* < 0.01). In addition, the MCI-amnestic group also showed increased whole-brain atrophy compared with nonamnestic group (*p* = 0.048). Consistent with the PBVC comparisons, there was a main effect of group on ventricular enlargement [F(2,130) = 7.1, *p* < 0.01; eta^2^ = 0.10]. The PD-MCI group showed increased percentage of ventricular enlargement relative to controls (*p* = 0.04) and PD-NC (*p* < 0.01). Stratified comparisons at baseline also showed that only the MCI-amnestic group had greater percentage of ventricular enlargement relative to healthy controls (*p* = 0.016) and PD-NC (*p* < 0.01).

Regarding the comparisons between PD_converters_ and PD_stable_, there were no significant differences in age, gender, education, UPDRS, and duration of illness between both PD_converters_ and PD_stable_. Although whole-brain atrophy was numerically greater in PD_converters_ (−0.5% ± 0.7%) compared with PD_stable_ (−0.4% ± 0.5%), this difference was not significant (Student *t* test; *p* = 0.44, Cohen's d = 0.2). However, PD_converters_ showed a significant increase in ventricular enlargement over 18 months compared with the PD_stable_ group (Student *t* test; *p* = 0.015, Cohen's d = 0.7; Supplementary Fig. 1).

### 3.3 Correlations of imaging outcome measures with clinical and cognitive functions

**Fig. 3:**
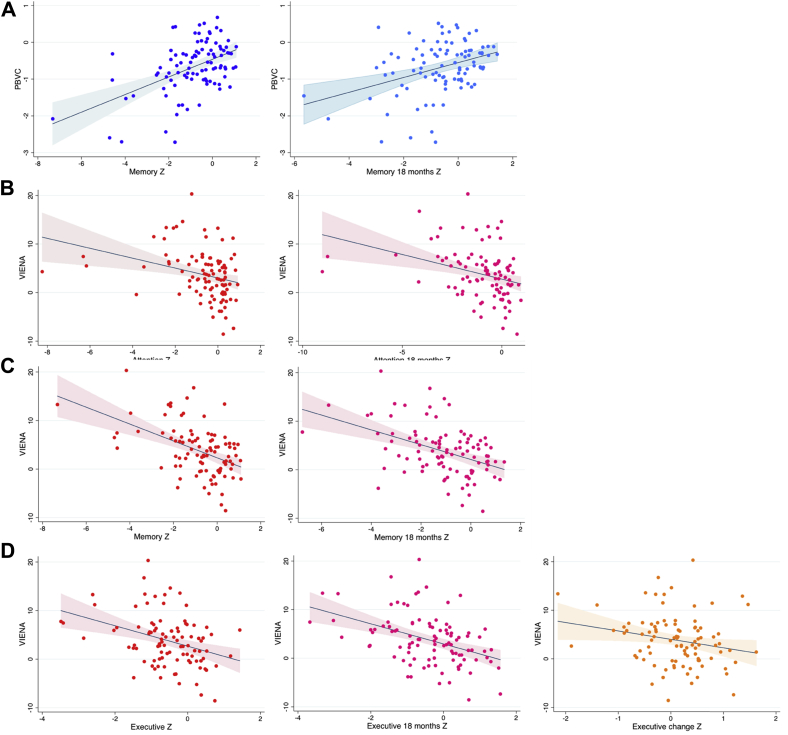
Scatter-plots showing the significant associations between whole-brain atrophy and ventricular enlargement with MDS cognitive domains in the total PD sample. (A) PBVC–memory, memory follow-up (B) VIENA–attention, attention follow-up (C) VIENA–memory, memory follow-up (D) VIENA–executive function, executive follow-up, and executive change. Abbreviations: MDS, Movement Disorders Society; PBVC, percentage brain volume change; VIENA, ventricular enlargement.

In the overall nondemented PD sample, we investigated the correlations of PBVC and ventricular enlargement over 18 months with global cognition and cognitive domains (Fig. 3). After accounting for age, gender, and LEDD, partial correlations revealed a positive association of PBVC with global cognition as assessed using the MoCA at baseline (r = 0.23, *p* = 0.03) as well as at follow-up (r = 0.24, *p* = 0.02), although there was no significant correlation with % change of MoCA change over 18 months (r = −0.1, *p* = 0.44). With respect to the MDS cognitive domains, PBVC were significantly associated with the memory domain at baseline (r = 0.38, *p* < 0.01) and follow-up (r = 0.24, *p* = 0.02), but not with change over 18 months (r = −0.15, *p* = 0.14).

Ventricular enlargement over 18 months was highly correlated with poorer MoCA at baseline (r = −0.35, *p* ≤ 0.01) and follow-up (r = −0.31, *p* < 0.01). It was also associated with poorer performance in various MDS cognitive domains at baseline, including attention (r = −0.25, *p* = 0.02), memory (r = −0.36, *p* < 0.01), and executive function (r = −0.22, *p* = 0.035). At 18 months, ventricular enlargement was also associated with attention (r = −0.21, *p* = 0.04), memory (r = −0.32, *p* < 0.01), executive function (r = −0.33, *p* < 0.01), and language (r = −0.21, *p* = 0.04). With respect to cognitive change over 18 months, ventricular enlargement was correlated with worsening executive function (r = −0.26, *p* = 0.01).

In terms of clinical features, UPDRS3 at follow-up was correlated with ventricular enlargement (r = 0.28, *p* = 0.01) and whole-brain atrophy (r = −0.20, *p* = 0.04) but not at baseline or change over time.

### 3.4 Correlations of global atrophy and ventricular enlargement with CSF markers

There were no significant differences in age, gender, MMSE, PBVC, and ventricular enlargement between the subset of PD patients with CSF samples (n = 35) compared with the rest of the PD group (n = 65), although disease duration was significantly shorter in those who underwent lumbar puncture (*p* < 0.01). The mean concentrations of the CSF measurements are described in Table 2.

**Table 2:** Demographics, clinical, and imaging characteristics of the subset of PD sample with CSF measurements

Subject groups	PD, No CSF	PD, CSF	*p* value
*n*	68	35	
PD-NC:PD-MCI	37:31	27:8	
Age (y)	65.4 ± 10	64.4 ± 9.6	0.6^c^
Age range	41.8–85.5	47.8–87.3	
Gender (male, %)	49, 72.1%	20, 57.1%	0.1^a^
Disease duration (mo)	25.7 ± 0.6	21.9 ± 0.5	<0.001^b^
UPDRS	27.3 ± 11.1	25.7 ± 10.8	0.6^c^
MMSE	28.6 ± 1.3	28.97 ± 1.0	0.2^b^
PBVC (%)	−0.73 ± 0.8	−0.58 ± 0.6	0.5^b^
Ventricular enlargement (%)	4.3 ± 6.6	3.90 ± 4.8	0.8^b^
CSF T-Tau (pg/mL)		130.1 ± 102.8	
CSF P-Tau (pg/mL)		48.1 ± 20.7	
CSF AB42 (pg/mL)		966.2 ± 300.0	
CSF AB40 (pg/mL)		10,968.3 ± 4610.0	
CSF AB42/AB40 ratio		0.10 ± 0.03	
CSF A-SYN (pg/mL)		92.4 ± 49.0	
	PD, No CSF	PD, CSF	*p* value
*n*	65	35	
PD-NC:PD-MCI	37:28	27:8	
Age (y)	65.2 ± 10.1	64.4 ± 9.6	0.7^c^
Age range	41.8–85.5	47.8–87.3	
Gender (male, %)	46, 71%	20, 57.1%	0.2^a^
Disease duration (mo)	25.8 ± 5.2	21.9 ± 3.1	<0.001^b^
UPDRS	27.1 ± 11.1	25.7 ± 10.8	0.5^c^
MMSE	28.6 ± 1.3	29.0 ± 1.0	0.2^b^
PBVC (%)	−0.72 ± 0.8	−0.58 ± 0.6	0.6^b^
VIENA (%)	4.2 ± 5.6	3.1 ± 4.3	0.3^t^
CSF T-Tau (pg/mL)		130.1 ± 102.8	
CSF P-Tau (pg/mL)		48.1 ± 20.7	
CSF Aβ42 (pg/mL)		966.2 ± 300.0	
CSF Aβ40 (pg/mL)		10,968.3 ± 4610.0	
CSF Aβ42/Aβ40 ratio		0.10 ± 0.03	
CSF *α*-synuclein (pg/mL)		92.4 ± 49.0	

Values expressed as mean ± SD.

Key: CSF, cerebrospinal fluid; MMSE, Mini–Mental State Exam, PBVC, percentage brain volume change; UPDRS, Unified Parkinson's Disease Rating Scale III; VIENA, ventricular enlargement.

a χD test.

b Wilcoxon rank-sum test.

c Student *t* test.

**Fig. 4:**
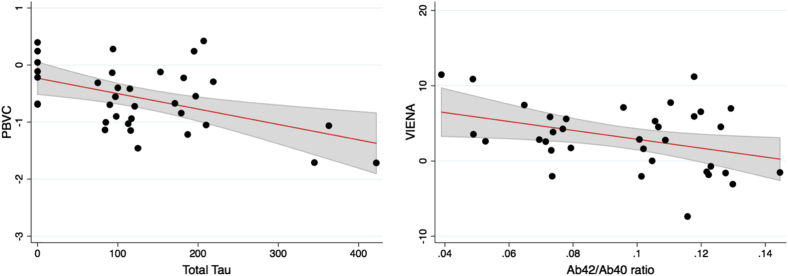
Scatter plots showing significant associations between CSF markers (total tau and Aβ42/Aβ40 ratio) at baseline and MRI change measures (PBVC and VIENA) respectively. Abbreviations: PBVC, percentage brain volume change; VIENA, ventricular enlargement.

We investigated the correlations of imaging outcome measures with CSF markers using partial correlation tests, accounting for age, gender, LEDD, and disease duration (Fig. 4). Increased whole-brain atrophy rates were associated with elevated baseline levels of T-Tau (r = −0.40, *p* = 0.03) but not with AB42/Ab40 ratio (r = 0.30, *p* = 0.11). Increased ventricular enlargement over 18 months was significantly correlated with a lower Aβ42/Aβ40 ratio (r = −0.38, *p* = 0.03), although a trend was observed with greater T-Tau levels (r = 0.35, *p* = 0.06). We did not find any significant correlations between CSF α-synuclein and PBVC (r = 0.12, *p* = 0.51) or ventricular enlargement (r = −0.13, *p* = 0.50).

Stratified correlations according to low and normal levels of CSF Aβ42 revealed that significant relationships between MRI and CSF markers were predominantly driven by the low Aβ42 group (Supplementary Fig. 1). T-Tau was correlated with PBVC (r = −0.67, *p* = 0.01) and VIENA (r = 0.62, *p* = 0.02), whereas Aβ42/Aβ40 ratio was associated with PBVC (r = 0.58, *p* = 0.04) and VIENA (r = −0.75, *p* < 0.01). Notably, these relationships were not found in the normal CSF Aβ42 group.

### 3.5 Associations of regional volumetric changes with CSF markers

**Fig. 5:**
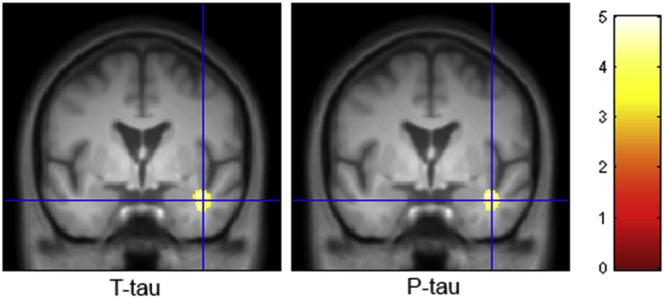
Correlations of T-Tau and P-Tau with volumetric changes over 18 months, covarying for age, sex, and LEDD. Overlay on average of the group scans in MNI space, crosshair at 41.5, 0, −25.5 mm. Thresholded at *p* < 0.001 uncorrected, min cluster = 200. Color bar represents *t* statistic. Abbreviations: LEDD, Levodopa equivalent daily dose. (For interpretation of the references to color in this figure legend, the reader is referred to the Web version of this article.)

We used tensor-based morphometry to further investigate the associations of CSF T-Tau and P-Tau with localized volumetric brain changes. There were trend-level clusters in the right temporal lobe white matter where increased CSF T-Tau (*p* = 0.065) and P-Tau (*p* = 0.055) correlated with tissue contraction over time (Fig. 5).

### 3.6 Sample size calculations

**Fig. 6:**
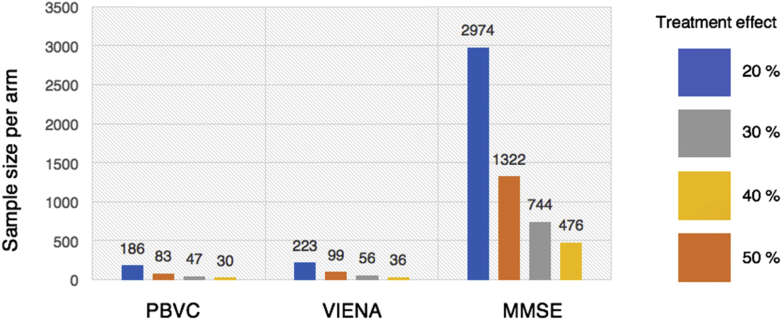
Necessary sample size per treatment arm using PBVC, VIENA, global cognitive decline in PD-MCI. Calculations were performed on the assumption of a treatment effect corresponding to 20%–50% reduction in the decline of the outcome measure, at 80% power, and a 2-sided 0.05 level of significance. Abbreviations: PBVC, percentage brain volume change; PD-MCI, Parkinson's disease with mild cognitive impairment; VIENA, ventricular enlargement.

Calculations for sample sizes are summarized in Fig. 6. Both whole-brain atrophy and ventricular enlargement required substantially smaller sample sizes compared with global cognitive decline (MMSE) for detecting significant differences over time in PD-MCI patients versus controls. To detect a 20% reduction, 186 and 223 patients per arm are required for whole-brain atrophy and ventricular enlargement respectively. In contrast, 2974 patients per arm are required to detect an equivalent degree of slowing of global cognitive decline. Sample size estimates were not derived for PD-NC because there were no significant differences compared with controls in both imaging measurements.

## 4 Discussion

### 4.1 Main findings

We performed a longitudinal study to investigate whole-brain atrophy and ventricular enlargement over 18 months in the ICICLE cohort of nondemented PD. First, MRI measurements of whole-brain atrophy and ventricular enlargement were significantly increased in the PD-MCI group, particularly in patients with memory impairment. Second, both global imaging markers were associated with several cognitive domains as well as CSF markers of AD pathology. Finally, sample size calculations showed that MRI measurements would be more sensitive than neuropsychological assessments to detect treatment effects in clinical trial. These collective findings provide reasonable support for the use of imaging measurements as surrogate markers in early interventional trials that target cognitive aspects of PD.

### 4.2 Increased whole-brain atrophy and ventricular enlargement in PD-MCI

The literature on progressive whole-brain atrophy in PD is still inconclusive. Increased atrophy rates in nondemented PD have been reported in 1 study (Hu et al., 2001), but not in others (Burton et al., 2005, Guevara et al., 2016). These differences are mostly ascribed to small sample sizes and heterogeneity in cognitive profiles, especially as PD-MCI was not distinguished from PD-NC. An earlier study that reported increased whole-brain atrophy rates in PD involved a relatively small sample of PD patients (n = 8) who were impaired in memory and visuospatial functions (Hu et al., 2001), both of which have been shown to herald subsequent progression to PD-D (Williams-Gray et al., 2009). In contrast, the PD groups in the negative studies were not cognitively impaired at baseline (Burton et al., 2005, Guevara et al., 2016). By stratifying our analyses according to the MDS criteria for PD-MCI, our observations of greater whole-brain atrophy may reconcile these inconsistencies and provide further support for the conceptualization of PD-MCI as a distinct clinical entity (Litvan et al., 2011)—a necessary prerequisite for enrichment of future clinical trials. Significant expansion of the ventricles over 18 months was demonstrated in PD-MCI compared with PD-NC and healthy controls. A previous 36-month study only found increased ventricular enlargement in PD-D, whereas nondemented PD did not differ from healthy controls (Camicioli et al., 2011). Together, these longitudinal findings also add to the limited body of cross-sectional evidence suggesting that ventricular enlargement may be an early feature in prodromal PD-D but not in PD-NC (Apostolova et al., 2010, Dalaker et al., 2011).

### 4.3 Amnestic PD-MCI is associated with greater neurodegeneration

Stratified analyses of the PD-MCI subgroups showed that whereas whole-brain atrophy and ventricular enlargement were significantly greater in the amnestic group, the nonamnestic subtype were not significantly different from healthy controls and PD-NC, although both imaging measures were intermediate between PD-NC and PD-MCI amnestic groups. These discrepancies may imply differential sites of pathology among various subtypes of PD-MCI (Janvin et al., 2006), such that PD-amnestic individuals are further along the path toward PD-D compared with their nonamnestic counterparts. Similarly, it has been suggested that certain cognitive domains are more predictive of dementia than others. The “Dual Syndrome Hypothesis” distinguishes a relatively stable fronto-striatal profile of impairment from “posterior cortical” deficits (i.e., memory) that are associated with more pronounced cholinergic deficits and increased progression to dementia (Kehagia et al., 2012). An important caveat is that 22/25 of our amnestic PD-MCI also had impairments in other domains (i.e., multi-domain impairment). Future studies with larger sample sizes in each group (single-nonamnestic/amnestic and multidomain-nonamnestic/amnestic) would be needed to clarify the neurodegenerative profile associated with each cognitive subtype of PD-MCI.

### 4.4 Ventricular enlargement is associated with conversion to PD-MCI

Longitudinal MRI studies may also be sensitive to early cognitive decline in PD-NC. Camicioli et al. showed that ventricular enlargement—measured using a volumetric approach—was present only in PD subjects who converted to PD-D over 36 months (Camicioli et al., 2011). Our findings further demonstrated that significant ventricular enlargement could be detected earlier in PD-MCI over a shorter follow-up of 18 months. Notably, the Cohen's *D* effect size for increased ventricular enlargement (*d* = 0.7) in PD-NC_converters_ relative to PD-NC_stable_ was substantially larger compared with whole-brain atrophy (*d* = 0.2), which did not differ significantly between both PD-NC subgroups. Interestingly, there is clinicopathological evidence to suggest that ventricular enlargement is more strongly related to neuropathological features of AD compared with whole-brain atrophy (Erten-Lyons et al., 2013). Increased ventricular change has also been reported in MCI patients who later converted to AD over 6 months (Nestor et al., 2008). The consistency of these findings, acquired using different approaches, may suggest that ventricular enlargement is a viable marker for prodromal neurodegeneration in PD.

### 4.5 Neuropsychological correlates of MRI measurements

The second objective of this study was to elucidate the clinical relevance of both global MRI measurements. Whole-brain atrophy was significantly associated with baseline and 18-month measurements of global cognition and memory, whereas ventricular enlargement showed additional correlations with multiple cognitive domains as well as motor features. These correlations are in broad agreement with previous studies in MCI and AD (Apostolova et al., 2010, Jack et al., 2004, O'Brien et al., 2001, Sluimer et al., 2008). Interestingly, ventricular enlargement was also associated with longitudinal changes in executive function, an early PD feature that is intimately linked with dopaminergic deficits in fronto-striatal networks (Foltynie et al., 2004, Janvin et al., 2005).

These findings may suggest that ventricular enlargement, in addition to being a sensitive state marker, shows promise in staging cognitive decline, at least for those modulated by fronto-striatal deficits in early nondemented PD. Several reasons could account for the closer coupling of ventricular enlargement to a multitude of clinical features, an observation that has also been reported in a longitudinal multiple sclerosis study using both SIENA and VIENA (the same imaging techniques employed in this study; Lukas et al., 2010) as well as another clinicopathological study in AD (Erten-Lyons et al., 2013). These include: (1) methodologically, the edge displacement technique could be more sensitive and accurate along the smoother ventricular edges (VIENA) compared with the convoluted cortical folds (SIENA; Lukas et al., 2010); (2) alternatively, ventricular enlargement may be a more dynamic measure over time as subtle reductions of brain volume could result in relatively larger expansions of the initially small ventricles (Anderson et al., 2006, Erten-Lyons et al., 2013, Lukas et al., 2010); and (3) topologically, ventricular enlargement could also be more functionally relevant in the context of PD. The lateral and third ventricles are in close proximity with subcortical structures, whereas the fourth ventricle is situated within the pons and medulla, both of which are regions preferentially vulnerable to the ascending progression of Lewy body pathology from the brainstem to the cerebral cortex (Braak et al., 2004).

### 4.6 Baseline CSF correlates of MRI measurements

The third objective of the study was to investigate these structural changes in relation to baseline CSF markers. Elevated CSF T-Tau levels, rather than Aβ42/Aβ40, were correlated with increased percentage of whole-brain atrophy. These findings are in broad agreement with the literature showing an association between CSF tau and brain morphological measures across PD (Beyer et al., 2013, Compta et al., 2012), MCI, and AD (Solé-Padullés et al., 2011, Thomann et al., 2009), and further implicate tau as an important component of neurodegeneration in PD.

In our study, the correlations between ventricular enlargement and lower AB42/AB40 ratios are consistent with previous findings looking at CSF (Beyer et al., 2013, Ott et al., 2010) and [^11^C]-PiB PET imaging studies (Sarro et al., 2016). In one of the largest MRI analyses of the ADNI cohort, Chou et al. showed that ventricular enlargement correlated strongly with decreased CSF Aβ42 across healthy controls, MCI, and AD. However, the association of CSF tau with ventricular enlargement was less robust as it did not persist after adjusting for covariates such as age (Chou et al., 2009). Similarly, we found that CSF tau was only tended to relate to ventricular enlargement. Ventricular enlargement, particularly of the temporal horn, could well be associated with adjacent atrophy in the medial temporal lobe—a preferential site of neurofibrillary tangle accumulation (Braak and Braak, 1995). In the absence of in vivo localization of tau pathology, we can only speculate that the CSF tau levels and measures of ventricular expansion may be regionally specific (i.e., confined to the temporal horn) and therefore lost when we consider global percentage change over time. This suggestion also fits with our tensor-based findings showing an association between CSF tau levels and atrophy in overlapping regions of the temporal lobe.

Could the relationships between CSF markers and MRI outcomes be influenced by amyloid status? As discussed, our study did not find an association between baseline CSF amyloid levels and longitudinal whole-brain atrophy although another study did find a posterior pattern of cortical thinning in PD subjects with low CSF Aβ42 levels (Compta et al., 2013). These findings are not necessarily in conflict with each other as a dichotomized amyloid status reflects a cumulative marker of AD pathology, such that PD subjects with low CSF Aβ42 values are more likely to be further on course to dementia. Methodological differences may also preclude direct comparisons as cortical thickness was investigated in the Compta et al. (2013) study. Indeed, there is evidence to suggest that cortical thickness has the greatest sensitivity to neurodegeneration in PD compared with other conventional structural MRI methods (Pereira et al., 2012). Interestingly, when we stratified the PD subjects according to a median split of CSF Aβ42, only the low CSF Aβ42 group showed a significant association with both whole-brain atrophy and ventricular enlargement and CSF tau and Aβ42/Aβ40 ratio (Supplementary Fig. 2). These findings also are in line with a recent tau PET study where the relationship between hippocampal [^18^F]-AV1451 uptake and volume was only significant in amyloid-positive subjects (Wang et al., 2016). These associations, confined to individuals with increased amyloid burden, collectively fit a model in which a pathological accumulation of amyloid is necessary before triggering a cascade of downstream neurodegeneration (Jack et al., 2013). The precise mechanisms for this process are still unknown, although there is evidence that β-amyloid may convert tau into its neurotoxic form, leading to cellular dysfunction and eventually cell death (Bloom, 2014).

### 4.7 CSF *α*-synuclein

The relationship between CSF *α*-synuclein levels and longitudinal atrophy has been relatively understudied (Campbell et al., 2015, Compta et al., 2014, Mattsson et al., 2013). In our present study, CSF *α*-synuclein was not associated with whole-brain atrophy or with ventricular enlargement. This runs counter to previous studies, where reductions in CSF *α*-synuclein have been shown to correlate with functional deficits in motor networks (Campbell et al., 2015) and frontal atrophy in nondemented PD (Compta et al., 2014). Interestingly, Compta et al. observed a reverse relationship in the PD-D group, where increased CSF *α*-synuclein was associated with atrophy in the parahippocampal gyrus and precuneus, both of which are commonly implicated in preclinical and established stages of dementia (Mak et al., 2017, Villemagne and Chételat, 2016). However, the correlations did not persist after adjusting for CSF tau, suggesting that any effects of CSF *α*-synuclein may be mediated through tau (Compta et al., 2014). In agreement with our study, a previous analysis involving the ADNI cohort also found that baseline levels of CSF *α*-synuclein were not correlated to atrophy brain rates across the spectrum of normal aging, MCI, and AD (Mattsson et al., 2013). These findings, considered together with autopsy-confirmed reports of relatively preserved brain structure in Lewy body diseases (Nedelska et al., 2015), suggest that the effects of *α*-synuclein pathology may be mediated via cellular dysfunction and functional network disruption (Peraza et al., 2015, Rittman et al., 2016). These are important questions that will be further addressed by the future development of PET radioligands for *α*-synuclein (Eberling et al., 2013).

### 4.8 Lack of correlations between imaging changes and cognitive decline

The breadth of findings in this study provides reasonable support for the use of imaging measures in clinical trials of nondemented PD. However, despite evidence of correlations between whole-brain atrophy and ventricular enlargement with *cross-sectional* cognitive domains and CSF markers, only ventricular expansion showed covariance with executive function over 18 months. This is surprising, as previous studies have reported correlations between both global MRI summaries and cognitive changes in PD-D and other neurodegenerative conditions (Camicioli et al., 2011, Jack et al., 2008, Knopman et al., 2009). Several explanations arise, including (1) the relatively stable trajectories or even minimal cognitive improvements in this PD cohort as well as other parkinsonian conditions (Guevara et al., 2016); (2) that the clinical assessments could fluctuate in response to other influences that are unrelated to disease progression; (3) greater cognitive reserve in highly functioning individuals may mask the underlying disease progression; (4) the length of follow-up could also be insufficient to detect cognitive decline, as other studies have reported negligible cognitive decline in nondemented PD subjects over 4 years (Aarsland et al., 2004); and (5) that our sample was evaluated while taking dopaminergic medication, which has been associated with both beneficial and adverse effects on executive function in PD (Cools et al., 2001, Molloy et al., 2006). This is by no means an exhaustive list of factors that could influence the variability in clinical and cognitive measurements, but it does highlight the value of using adjunctive imaging markers in clinical trials.

### 4.9 Impact of MRI outcomes on clinical trials

Consistent with other studies in MCI and AD (Jack et al., 2003, Ridha et al., 2008), we found that using cognitive testing (MMSE) as a trial end point would require significantly more patients to detect the same degree of treatment-related slowing of decline compared with MRI measures. Nevertheless, the effect size on a clinical scale may be differentially consequential compared to an equivalent change on MRI (i.e., a 20% reduction in MMSE decline may be more clinically meaningful compared with the same degree of reduced atrophy rates). Therefore, we recommend that neuroimaging should still be used in conjunction with cognitive assessments in trials designed to evaluate disease progression and the effectiveness of disease modifying treatments. Several studies have found that approximately 35–100 AD and 100–200 MCI patients are necessary to detect a 25% reduction in brain atrophy (Beckett et al., 2010, Ho et al., 2010, Hua et al., 2010, Nestor et al., 2008). Our findings extend these reports to PD-MCI, a condition for which there are currently no disease-modification trials. Assuming a clinical trial design with 18 months of follow up and a 20% absolute reduction in volume loss, the required sample sizes for whole-brain atrophy (n = 186) and ventricular enlargement (n = 223) are larger than previous estimates in AD (n = 115; Fox et al., 2000), in line with the milder atrophy seen in PD or PD-D relative to AD (Beyer et al., 2007, Burton et al., 2004, Mak et al., 2015a). Nevertheless, these numbers are not prohibitive in terms of logistics and cost, particularly for secondary preventive trials in PD-MCI patients. Furthermore, the relationships of both imaging outcomes with AD pathologies—not alpha-synuclein—suggest that patients with Lewy body diseases might stand to benefit from interventions directed against amyloid and tau pathologies. This will no doubt be a topic for important validation using in vivo PET imaging of amyloid and tau in Lewy body diseases (Gomperts et al., 2016, Kantarci et al., 2017, Sarro et al., 2016).

### 4.10 Strengths and limitations

The main strength of this prospective cohort study is its longitudinal follow-up of an incident cohort allowing correlation of longitudinal global atrophy in PD with cognitive function. The comprehensive analyses of CSF enabled the investigation of the differential correlation of each CSF marker to longitudinal atrophy, an area of research that is currently understudied in PD. We used validated approaches to measure longitudinal global changes which reduces inter-subject variability as each subject serves as his or her own control (Smith et al., 2007, Vrenken et al., 2014). There are several practical benefits associated with global summaries particularly in the context of large-scale clinical trials. These measurements are fully automated, fast (20–30 minutes per subject), and they have been shown to demonstrate high accuracy, high test-retest reliability as well as robustness against different scanner sequences (Smith et al., 2002). Compared with regional measurements (e.g., bilateral hippocampus), the lateral ventricle is the most consistently measured structure cash (Cash et al., 2015). This reduction in variability is crucial for reducing sample sizes necessary to detect a treatment effect. Several potential limitations should be recognized. Subjects were assessed while taking their medication, which could influence cognitive function and CSF measures. To address this, we accounted for LEDD in all group comparisons and correlational analyses. In the cognitive battery we used, our assessment of visuospatial function was limited as we only had 1 representative test. However, the pentagon copying item of the MMSE has a high predictive value for the development of dementia in PD (Garcia-Diaz et al., 2014, Williams-Gray et al., 2007). In addition, the magnitude of the correlations between imaging measures and cognitive domains were rather modest despite the statistical significance. Many factors contribute to cognitive decline. Some but not all of these will be expressed through differences in brain structure, in addition to which the imaging markers represent a greatly reduced dimensionality of patients' neural systems. Correction for multiple comparisons was not performed in this study due to the intercorrelated nature of the MDS cognitive domains and should be replicated in independent cohorts. The extent and accuracy of longitudinal imaging measurements may be influenced by baseline volumes too. For instance, ventricular enlargement may be greater early in the disease when the baseline volume is smaller. However, the methods used in this paper are solely longitudinal, providing the amount of % change between scans but not the initial volume at baseline (Vrenken et al., 2014). While SIENA/VIENA do not permit cross-sectional volumetric quantification, they use consecutive images at 2 time-points to estimate the local displacements between edges from serial images. This measurement has been shown to demonstrate high accuracy, high test-retest reliability as well as robustness against different scanner sequences (Smith et al., 2002). Furthermore, the accuracy of VIENA is not affected by the initial ventricular volume (manually segmented; Vrenken et al., 2014). In addition to greater processing time and demands, volume-based quantification typically require MR images with good intensity contrasts between gray and white matter, which may be decreased in aging. Such requirements may not always be satisfied in large-scale clinical trials or are unavailable in retrospective studies from archived images.

## 5 Conclusion

In summary, we found that global atrophy and ventricular enlargement, estimated using fully automated and less labor-intensive approaches are sensitive to disease progression by way of group-differences between PD-NC and PD-MCI. These MRI markers could be used to enrich for patient cohorts for clinical trials of disease modifying therapies as more aggressive profiles of whole-brain atrophy and ventricular enlargement are associated with individuals who are more likely to demonstrate a significant cognitive decline over the course of a clinical trial. Indeed we have further shown that using both global MRI markers would require much smaller sample sizes for trials compared with neuropsychological assessments to detect an equivalent degree of treatment effects. However further studies are needed to show the utility of this approach using independent cohorts of patients.

## Disclosure statement

Elijah Mak, Su Li, Guy Williams, Michael Firbank, Gordon Duncan, Adrian Owen, Tien Khoo, and David Brooks have no competing interests. Rachael Lawson is supported by grants from the Lockhart Parkinson's Disease Research Fund. Alison Yarnall is funded by the Biomedical Research Unit, Newcastle University and has previously been supported by grants from the Lockhart Parkinson's Disease Research Fund and the Michael J. Fox Foundation. She has received honoraria from Teva-Lundbeck and sponsorship from Teva-Lundbeck, UCB, GlaxoSmithKline (GSK), Genus, Britannia Pharmaceuticals Ltd and AbbVie for attending conferences. Brit Mollenhauer has received independent research grants from TEVA-Pharma, Desitin, Boehringer Ingelheim, GE Healthcare, and honoraria for consultancy from Bayer Schering Pharma AG, Roche, AbbVie, TEVA-Pharma, Biogen and for presentations from GlaxoSmithKline, Orion Pharma, TEVA-Pharma and travel costs from TEVA-Pharma. She is a member of the executive steering committee of the Parkinson Progression Marker Initiative of the Michael J. Fox Foundation for Parkinson's Research and has received grants from the BMBF, EU, Deutsche Parkinson Vereinigung, Michael J. Fox Foundation for Parkinson's Research, Stifterverband für die deutsche Wissenschaft and has scientific collaborations with Roche, Bristol Myers Squibb, Ely Lilly, Covance and Biogen. David Burn has received grants from NIHR, Wellcome Trust, and Parkinson's UK. He has received speaker fees from Acadia Pharmaceuticals. Roger Barker has grants from NIHR, EU, Parkinson's UK, CPT, Rosetrees Trust. He receives editorial monies from Springer and royalties from Wiley. James Rowe reports grants from Wellcome Trust, Medical Research Council, NIHR, and from Parkinson's UK. John O'Brien reports grants from the Medical Research Council, NIHR, ARUK and the Alzheimer's Society and has acted as a consultant for GE Healthcare, Lilly, TauRx and Cytox.
