# Supplementary material for: Longitudinal whole-brain atrophy and ventricular enlargement in nondemented Parkinson's disease
Source: Neurobiol Aging. 2017 Jul;55:78–90. doi: 10.1016/j.neurobiolaging.2017.03.012 (PMC5454799; doi:10.1016/j.neurobiolaging.2017.03.012)
Supplement: Supplementary Figures 1 and 2 [file mmc1.docx]

**SUPPLEMENTARY MATERIAL**

**
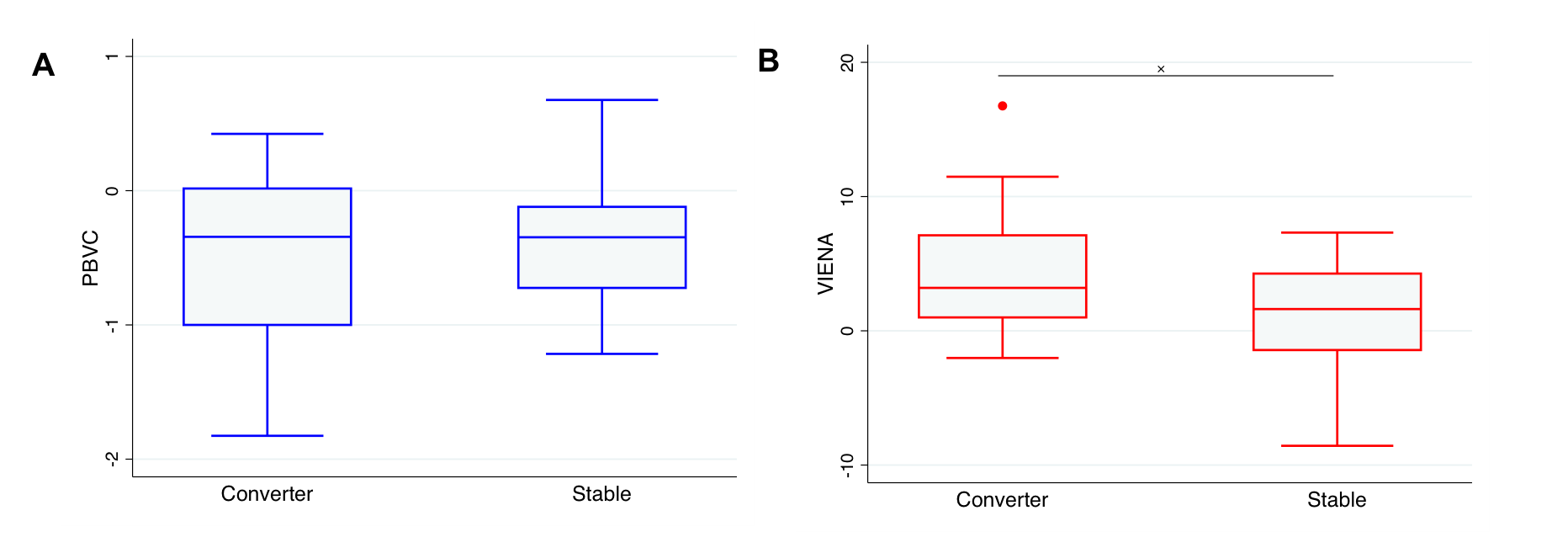
**

***Supplementary Figure 1.*** Subgroup analyses of whole brain atrophy and ventricular enlargement in PD-NC_converters_ and PD-NC_stable._ A – B: PBVC and VIENA in PD-NC_converters_ compared to PD-NC_stable_. Abbreviations: PBVC = Percentage brain volume change; VIENA = Ventricular enlargement.


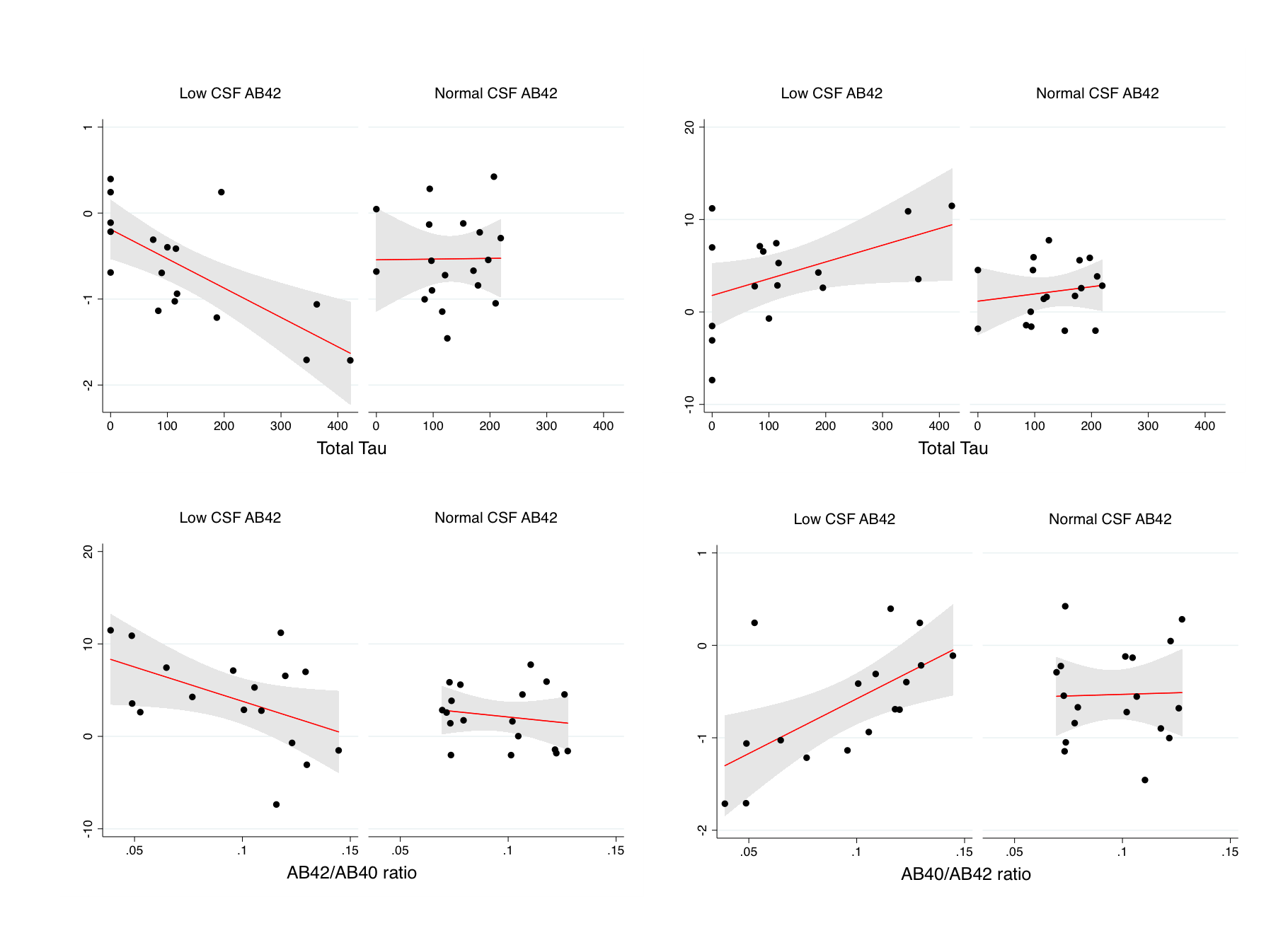


**Supplementary Figure 2.** Scatter plots showing significant associations between CSF markers (Total Tau and Aβ42 / Aβ40 ratio) at baseline and MRI change measures (PBVC and VIENA) respectively**.** The sample of PD subjects with CSF measurements were stratified according to low and normal Aβ42 groups using a median split. Abbreviations: PBVC = Percentage brain volume change; VIENA = ventricular enlargement.
